# Relative blood volume changes during haemodialysis estimated from haemoconcentration markers

**DOI:** 10.1038/s41598-020-71830-0

**Published:** 2020-09-09

**Authors:** L. Pstras, J. Waniewski, A. Wojcik-Zaluska, W. Zaluska

**Affiliations:** 1 Nalecz Institute of Biocybernetics and Biomedical Engineering, Polish Academy of Sciences, Trojdena 4, 02-109 Warsaw, Poland; 2 Department of Physical Therapy and Rehabilitation, Medical University of Lublin, Lublin, Poland; 3 Department of Nephrology, Medical University of Lublin, Lublin, Poland

**Keywords:** Renal replacement therapy, Biomedical engineering, Computer modelling

## Abstract

Relative blood volume (RBV) monitoring is frequently used in haemodialysis patients to help guide fluid management and improve cardiovascular stability. RBV changes are typically estimated based on online measurements of certain haemoconcentration markers, such as haematocrit (HCT), haemoglobin (HGB) or total blood protein concentration (TBP). The beginning of a haemodialysis procedure, i.e. filling the extracorporeal circuit with the patient’s blood (with the priming saline being infused to the patient or discarded) may be associated with relatively dynamic changes in the circulation, and hence the observed RBV changes may depend on the exact moment of starting the measurements. The aim of this study was to use a mathematical model to assess this issue quantitatively. The model-based simulations indicate that when the priming saline is not discarded but infused to the patient, a few-minute difference in the moment of starting RBV tracking through measurements of HCT, HGB or TBP may substantially affect the RBV changes observed throughout the dialysis session, especially with large priming volumes. A possible overestimation of the actual RBV changes is the highest when the measurements are started within a couple of minutes after the infusion of priming saline is completed.

## Introduction

One of the main goals of dialysis therapy is to remove from the patient the excess fluid that accumulates in the body due to kidney failure. In haemodialysis (HD) the excess fluid (water and small solutes) is removed from the blood flowing through the dialyzer in the process called ultrafiltration. The resulting blood volume reduction is compensated by the so called vascular refilling^1–4^, which consists in the absorption of fluid from the tissues in the capillary beds (driven mainly by the oncotic pressure difference across the capillary walls) combined with the ongoing lymphatic absorption at the whole-body level.

In a typical HD session, a few litres of fluid (2–4 L) are removed from the circulation over the course of a few hours (3–5 h), and hence, even with a properly working vascular refilling mechanism, the total blood volume is usually reduced. In order to maintain cardiovascular stability and sustain adequate organ perfusion, the reduction of the total blood volume cannot be excessively large or abrupt, otherwise the cardiovascular regulatory mechanisms may not be able to maintain sufficient blood pressure leading to intradialytic hypotension (IDH)—the most common complication of HD^5–9^. One of the means of preventing IDH is to monitor the blood volume changes and to provide an adequate reaction once the blood volume falls too low or too quickly (e.g. by a manual or automatic reduction of dialyzer ultrafiltration).

Given that it is not feasible to continuously or frequently measure the actual total blood volume during HD, in clinical settings the relative blood volume (RBV) changes are assessed based on changes in certain blood parameters, such as haematocrit (HCT), haemoglobin (HGB) or total blood protein concentration (TBP), which can be estimated from non-invasive measurements of optical, acoustic or other properties of blood flowing through the dialysis circuit^10,11^.

For instance, the RBV changes can be calculated from HCT variation using the following equation:

1 $$\Delta BV_{HCT} (t) = \left( {\frac{{HCT_{0} }}{{HCT_{t} }} - 1} \right) \times 100\,\,\,\,[\% ]$$

where at any time point t, the current level of haematocrit (HCT_t_) is compared to the initial reference haematocrit (HCT_0_).

**Figure 1:**
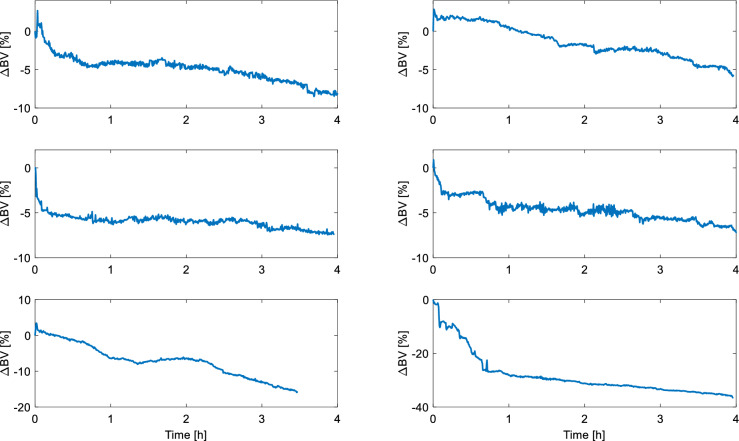
Examples of relative blood volume changes (∆BV) recorded by Crit-Line system (Fresenius Medical Care AG & Co, Bad Homburg, Germany) during maintenance haemodialysis (all curves start at 0%). The data comes from patients of Medical University of Lublin, Poland.

**Figure 2:**
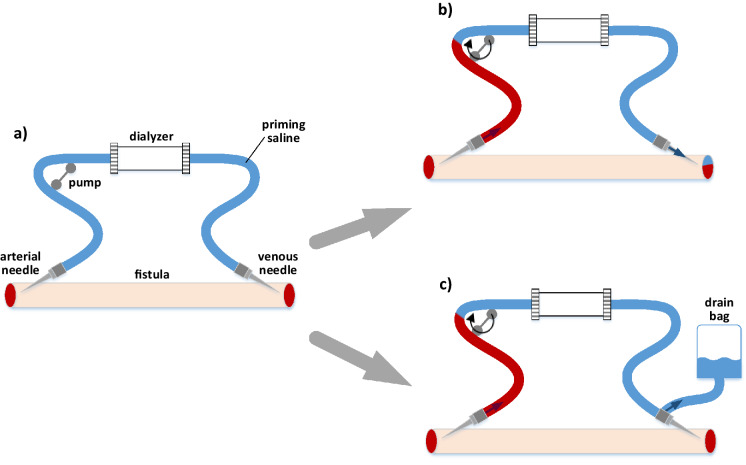
(**a)** Extracorporeal haemodialysis circuit filled with the priming fluid (saline). (**b)** Filling the extracorporeal circuit with the patient’s blood: the case with the priming fluid infused to the patient; (**c)** Filling the extracorporeal circuit with the patient’s blood: the case with the priming fluid discarded into the drain bag.

Even though the accuracy of monitoring RBV changes using the above methods to describe absolute blood volume changes is still subject to debate^12–15^, RBV monitoring is widely used in dialysis units (mainly with HCT as a haemoconcentration marker). The RBV monitors are either integrated into the dialysis machine (e.g. Blood Volume Monitor, Fresenius Medical Care AG & Co) or constitute a separate device installed in the dialysis circuit (e.g. Crit-Line, Fresenius Medical Care AG & Co). In both cases, the process of monitoring RBV changes is usually initiated manually by pressing a special button on the device. There is no universal procedure on when exactly such measurements should be started. For instance, the Crit-Line IV quickstart guide^16^ reads that, once the extracorporeal circuit is primed with blood (as per dialysis unit procedure) and the Crit-Line system is powered on and duly inspected, one should “wait 3–5 min with the blood pump at ≥ 150 mL/min” (to ensure that the priming saline residuals from the arterial blood chamber are fully mixed with blood and that the measured haematocrit is accurate and stable^17^). If the blood volume was changing (decreasing) steadily from the beginning of the HD session, a few-minute difference in terms of starting the RBV measurements would be rather negligible. However, in reality the blood volume (as estimated by RBV monitors, such as Crit-Line) rarely decreases linearly throughout the whole HD (see Fig. 1). Moreover, at the beginning of HD the blood volume changes detected by RBV monitors are often quite dynamic with relatively sudden drops or temporary increases in apparent blood volume (see Fig. 1), especially when the priming fluid, i.e. the fluid filling the extracorporeal circuit before dialysis, is not discarded but infused to the patient, when the circuit is filled with the patient’s blood, as in a typical priming protocol in U.S. dialysis units^18^ (see Fig. 2).

In light of the above, the aim of this paper was to use a mathematical model to analyse the importance of timing of RBV measurements during HD. In particular, we wanted to use model-based simulations to estimate how the difference in the starting moment of the measurements may affect the level of RBV changes indicated by the RBV monitor at the end of a dialysis session.

## Methods

The simulations of HD were performed using our earlier lumped-parameter compartmental model of cardiovascular response to haemodialysis^19^. The model integrates: (1) the cardiovascular system with baroregulation^20,21^, (2) the whole-body water and solute transport, (3) the extracorporeal circuit with a dialyzer. The model describes a non-pulsatile two-phase blood flow (plasma with suspended red blood cells) across 9 cardiovascular compartments (large arteries, small arteries and arterioles, systemic capillaries, small veins and venules, large veins, right heart, pulmonary arteries, pulmonary veins, left heart) and 3 extracorporeal compartments (arterial tubing, dialyzer, venous tubing) connected via an arteriovenous fistula. The baroreflex regulation consists of four mechanisms controlling heart rate, cardiac strength, peripheral resistance and venous unstressed volume in response to blood pressure measured by virtual baroreceptors localised in large arteries (high pressure baroreceptors) and right heart (low pressure baroreceptors). The model includes the transport of water and solutes between the capillaries and tissues at the whole-body level (across the capillary walls), between the tissues and body cells (across the cellular membrane) and between plasma and red blood cells within each cardiovascular compartment (across the red blood cell membrane). The analysed solutes include ions (sodium, potassium, chloride, bicarbonate and other cations and anions treated collectively), small solutes (urea and creatinine – standard markers of dialysis adequacy) and proteins (albumin and globulins). The model includes also the lymphatic system with the aggregated lymph flow from the tissue compartment to the large veins compartment. The model takes into account the different level of HCT between microcirculation and macro vessels and allows for dynamic changes of HCT within all circulatory compartments according to the water and solute transport processes induced by HD. For a full description of the model structure, equations and assumptions please see our earlier work^19^.

The model enables a detailed investigation of changes in pressure and volumes of all analysed compartments as well as changes in certain blood parameters, such as HCT, HGB or TBP, during a variety of HD scenarios. In this paper we analysed a typical 4-h HD session with 3 L of excess fluid removed at a constant ultrafiltration rate. The model accounts for filling of the extracorporeal circuit with the patient’s blood at the assumed rate of 100 mL/min with two cases considered: (1) the priming fluid infused to the patient (during this process the blood becomes diluted, but the amount of blood within the body remains roughly unchanged), (2) the priming fluid discarded to the drain bag (the total volume of blood within the patient’s body is reduced by the amount of blood used for filling the extracorporeal circuit). In the first case, the total ultrafiltration was always increased by the priming volume in order to keep the net ultrafiltration at 3 L. The priming fluid was assumed to be a normal saline (Na^+^ 154 mmol/L, Cl^-^ 154 mmol/L). The assumed volume of the arterial blood lines, venous blood lines and the dialyzer itself were 60, 60 and 100 mL yielding a total priming volume of 220 mL. A smaller and larger priming volumes of 160 and 280 mL were also analysed separately. Once the virtual extracorporeal circuit is filled with the patient’s blood, the model simulation is run for 2 min with the dialyzer pump rate gradually increasing to the target dialyzer blood flow rate of 300 mL/min (during this time the medical personnel usually checks the connections of blood lines and adjusts the dialyzer settings).

The analysis concerns a reference dialysis patient as defined in our previous work^19^ with the total pre-dialysis blood volume 5.1 L, central haematocrit 35%, mean corpuscular volume of red blood cells 90 fL, and the total body water 44.2 L. The blood flow rate through the fistula was set in the model to 950 mL/min and the cardiac output of 6.2 L/min was assumed. The pre-dialysis levels of plasma solutes were assumed as follows: Na^+^ 140 mmol/L, K^+^ 5 mmol/L, Cl^-^103 mmol/L, HCO3^-^ 22 mmol/L, other cations (Ca^2+^, Mg^2+^) 2 mmol/L, urea 27 mmol/L, creatinine 1 mmol/L, total protein 6.4 g/dL. The pre-dialysis blood haemoglobin was 11.9 g/dL. For all other model parameters, including the initial pressures and volumes of individual compartments, please see our previous work^19^.

The concentrations of ions in the dialysate fluid were set at the typical level with respect to the assumed plasma concentrations (Na^+^ 142 mmol/L, K^+^ 2 mmol/L, Cl^−^ 108 mmol/L, HCO3^−^ 34 mmol/L, other cations (Ca^2+^, Mg^2+^) 2 mmol/L. The dialyzer clearance of urea and other solutes was assumed at 210 mL/min, except for creatinine (168 mL/min) and bicarbonate ions (105 mL/min)^19^.

## Results

**Figure 3:**
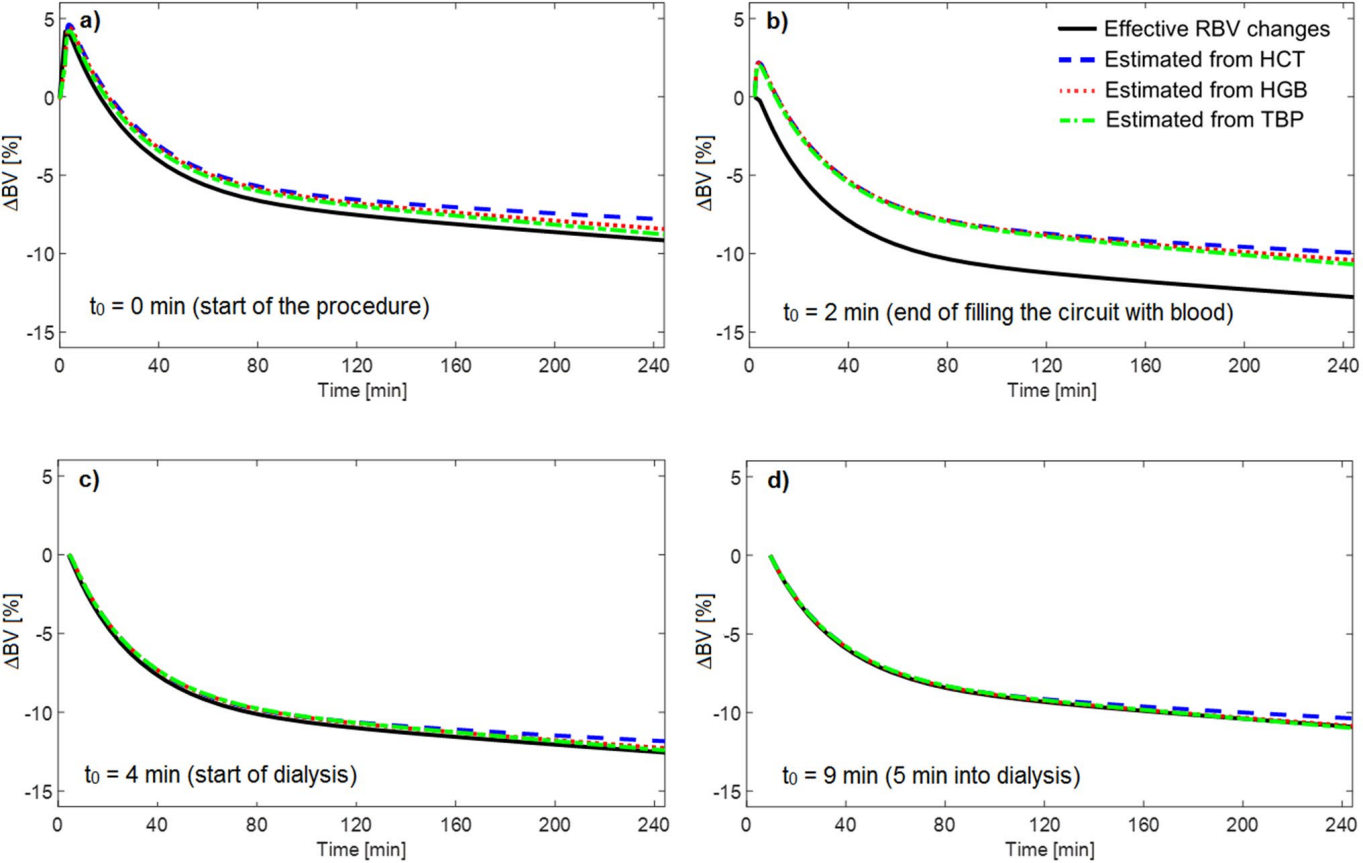
Relative blood volume changes (∆BV) during haemodialysis procedure (with the priming saline infused to the patient) as simulated by the model (solid line) and estimated from different model variables: central haematocrit (dashed line), whole blood haemoglobin concentration (dotted line), total blood protein concentration (dot-dashed line) corresponding to four different starting points of measurements: a) at the beginning of the whole haemodialysis procedure, i.e. at the start of filling the extracorporeal circuit with the patient’s blood; b) at the end of filling the extracorporeal circuit with the patient’s blood (ca. 2 min later); c) at the start of the actual haemodialysis (ca. 4 min later); d) 5 min into haemodialysis session (ca. 9 min later).

The RBV changes during the HD procedure (with the priming saline infused to the patient) simulated by the model and estimated from different blood parameters—HCT, HGB or TBP—measured in the virtual arterial blood line compartment are shown in Fig. 3 with four different starting points of measurements: (a) at the beginning of the whole HD procedure, i.e. at the start of filling the extracorporeal circuit with the patient’s blood; (b) at the end of filling the extracorporeal circuit with the patient’s blood (ca. 2 min later); (c) at the start of the actual HD (ca. 4 min later); (d) 5 min into haemodialysis session (ca. 9 min later). The RBV decrease simulated by the model at the end of HD was 9.2%, 12.8%, 12.6%, 10.8%, for cases (a), (b), (c), and (d), respectively. The corresponding RBV changes estimated by changes in HCT were 7.8%, 10.0%, 11.8%, 10.4%. In all cases, the RBV changes estimated from HCT variation were slightly lower than estimated from HGB or TBP, which is due to dialysis-induced osmotic water shift from erythrocytes to plasma that leads to a slight reduction in erythrocyte volume (please see Discussion).

**Figure 4:**
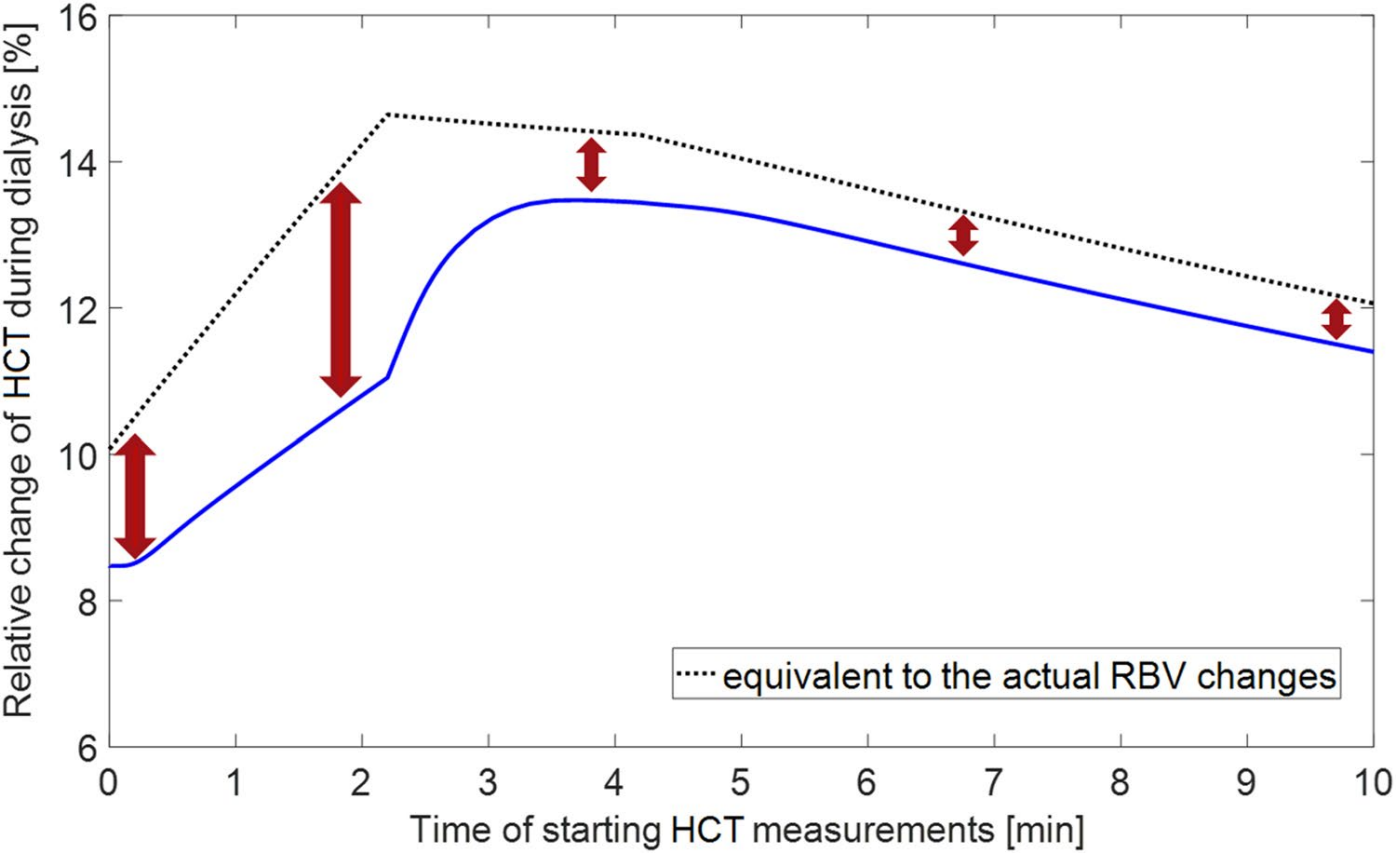
Model-simulated relative change of apparent central haematocrit (HCT) induced by a 4 h haemodialysis procedure (with the priming saline infused to the patient) as a function of the time of starting the measurements with respect to the beginning of the procedure (solid line) compared with virtual HCT changes equivalent to the actual relative blood volume (RBV) changes simulated by the model from a given moment of time (dotted line).

Figure 4 shows the simulated dialysis-induced relative changes of apparent central HCT (as would be seen by the HCT-measuring device in the dialyzer arterial tubing at the end of dialysis session) depending on the time of starting HCT measurements with the values varying from 8.5% to 13.5%. Regardless of the moment of starting the measurements, the HCT-based estimate of RBV changes during HD consistently underestimates the actual RBV changes simulated by the model with the maximal difference corresponding to the case when the measurements are started once the extracorporeal circuit is filled with the patient’s blood, i.e. circa 2.2 min after the beginning of the filling procedure. Note that beyond the time shown in Fig. 4, the relative change of HCT continues its linear decrease towards 0, as the moment of starting the HCT measurements approaches the end of HD.

**Figure 5:**
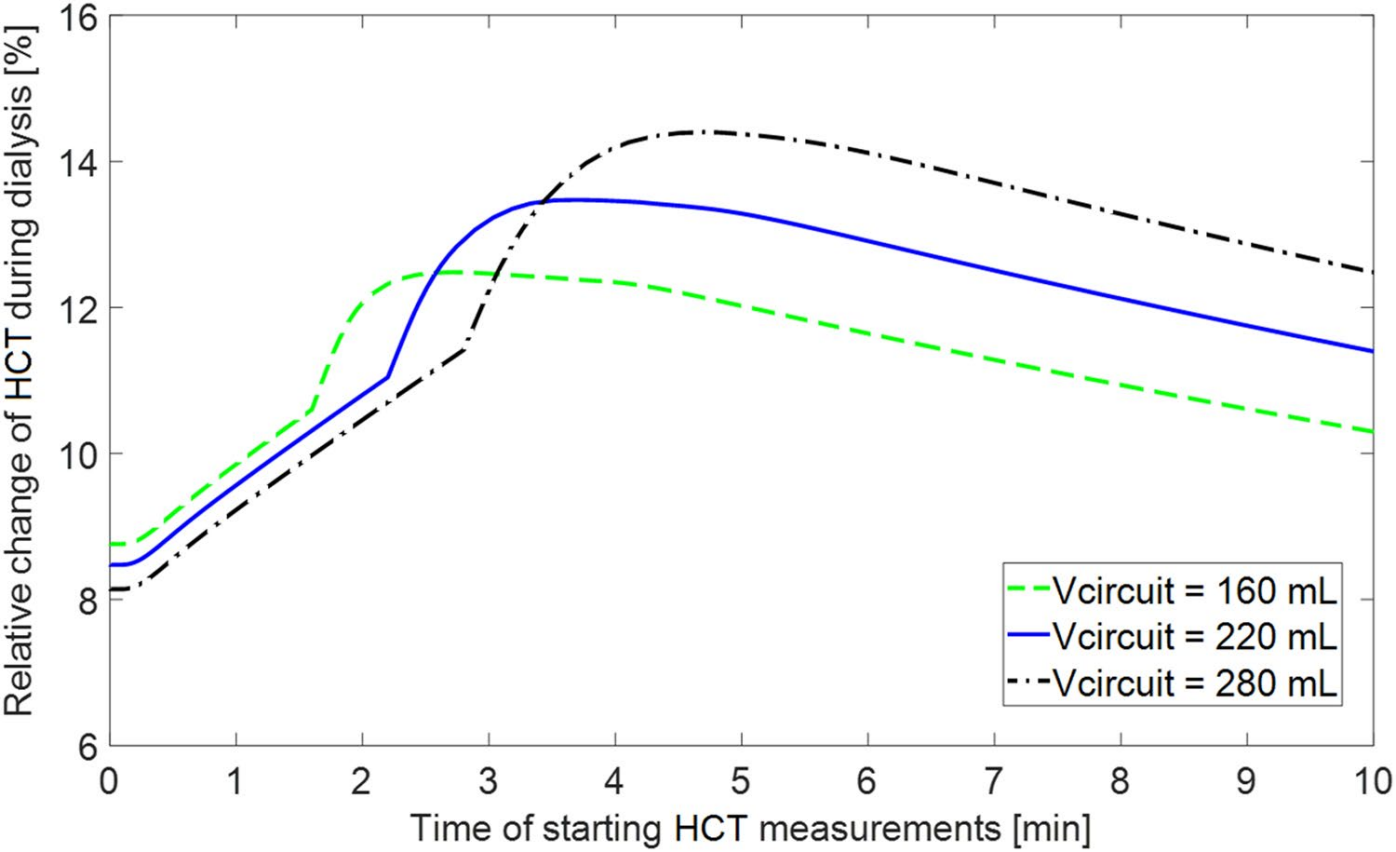
Model-simulated relative change of apparent central haematocrit (HCT) induced by a 4 h haemodialysis procedure (with the priming saline infused to the patient) as a function of the time of starting the measurements with respect to the beginning of the procedure for three different total priming volumes of the extracorporeal circuit.

As shown in Fig. 5, for a larger extracorporeal circuit with the total volume of 280 mL the corresponding differences in the apparent relative HCT change depending on the starting moment of the measurements are accordingly higher (relative HCT change at the end of HD varying between 8.1% and 14.4%). For a lower priming volume (i.e. a smaller volume of the extracorporeal circuit) the apparent dialysis-induced relative HCT change varies from 8.8% to 12.4% depending on the moment of starting the measurements.

**Figure 6:**
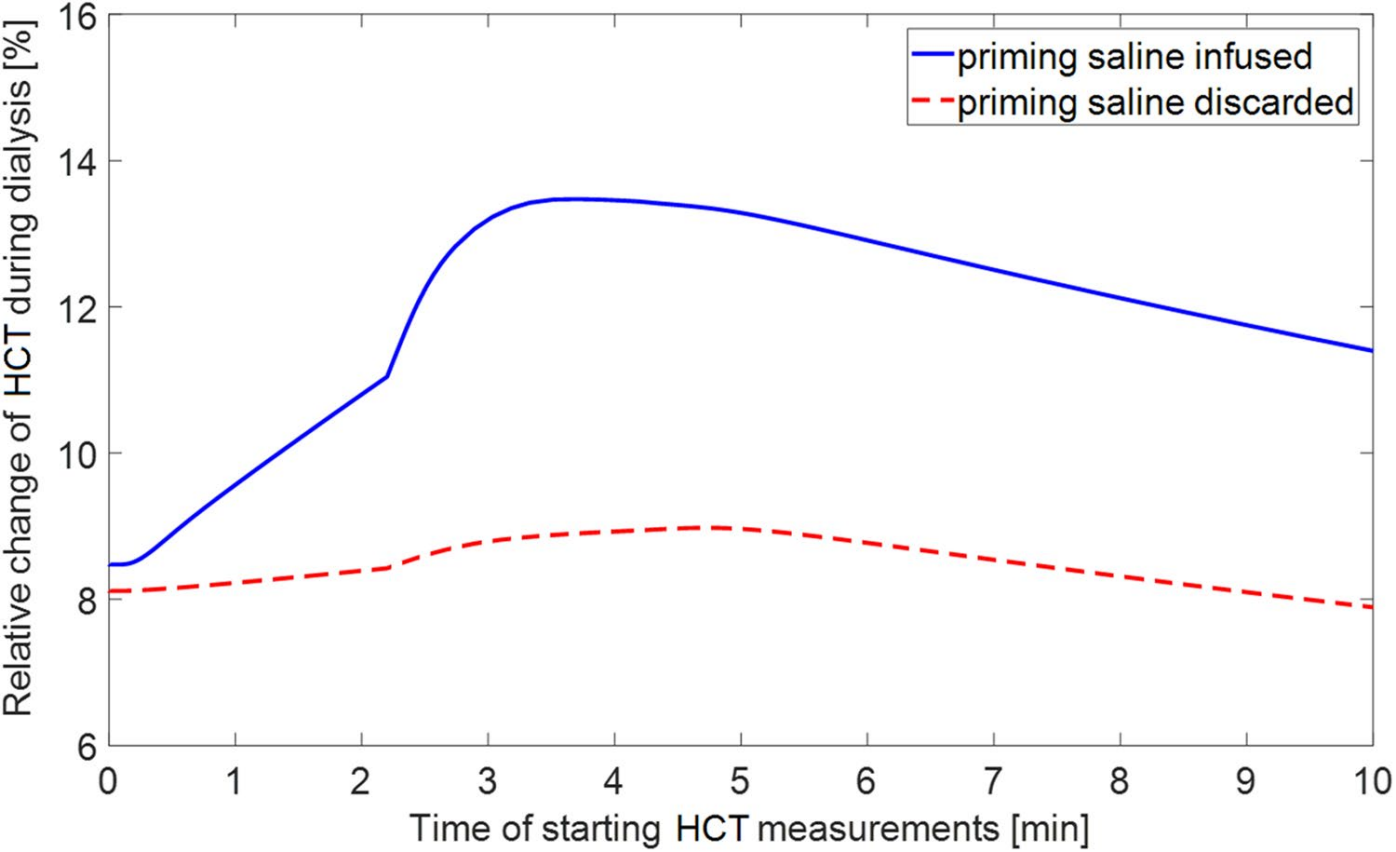
Model-simulated relative change of apparent central haematocrit (HCT) induced by a 4 h haemodialysis procedure as a function of the time of starting the measurements with respect to the beginning of the procedure for two cases: the priming saline infused to the patient (solid line) and the priming saline discarded (dashed line).

In the case when the priming saline was not infused to the patient but discarded, the analogous differences in model-simulated relative change of apparent HCT at the end of HD depending on the starting point of measurements were much smaller (below 1 percentage point) with the values of relative HCT change varying between 8.1% and 8.9% (see Fig. 6).

**Figure 7:**
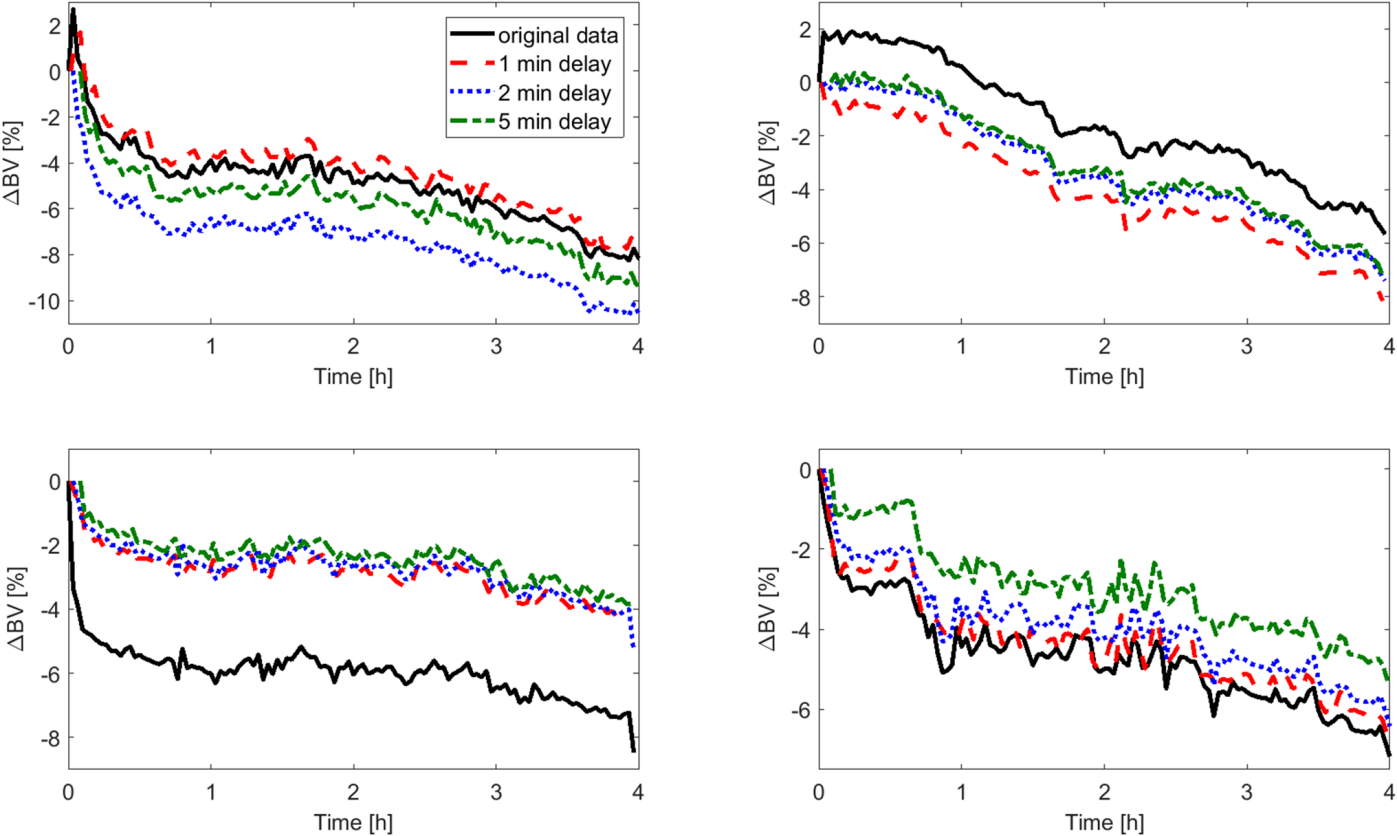
Examples of relative blood volume changes (∆BV) recorded by Crit-Line system (Fresenius Medical Care AG & Co, Bad Homburg, Germany) during maintenance haemodialysis in four different patients (black solid lines) compared with the calculated ∆BV curves that would be provided by the Crit-Line system if the measurements were started with a delay of 1 min (red dashed lines), 2 min (dotted blue lines), or 5 min (green dash-dotted lines). All curves start at 0%. The data comes from patients of Medical University of Lublin, Poland.

In addition to the presented model-based simulations, in Fig. 7 we show a few real-world examples of how the RBV estimated by the Crit-Line monitor would change if the measurements were started 1, 2 or 5 min later (the examples correspond to the first four cases presented in Fig. 1 with the data sampled at 1 min intervals for the sake of figure clarity). Please note that in the shown examples the original HCT measurements were initiated roughly at the beginning of the HD procedure (with the priming saline being infused to the patient), but unfortunately we were not able to determine retrospectively the exact moment of starting the measurements, which precluded us from a direct comparison with our model predictions. Nevertheless, these examples further underline the importance of the moment of initiating the measurements of haemoconcentration markers for estimating dialysis-induced RBV changes.

## Discussion

Our simulations show that if the priming saline is infused to the patient when the extracorporeal circuit is filled with the patient’s blood, the exact moment of starting the measurements of HCT changes in the arterial blood line has a significant impact on the obtained estimates of RBV changes during HD. This is due to the fact that at the beginning of the HD procedure, when the priming saline is infused to the patient, the total circulating blood volume is relatively quickly increased and the blood becomes diluted. The measurements of any haemoconcentration marker (i.e. HCT, HGB or TBP) started at the beginning of the whole procedure will have as a reference the undiluted ‘original’ patient’s blood. However, the later the measurements are started, the more diluted blood is taken as initial reference, and hence the higher RBV decrease is perceived during dialysis. After circa 4 min from the beginning of the whole procedure, the blood circulating within the body and across the extracorporeal circuit is uniformly mixed and starts to become haemoconcentrated (initially due to increased capillary filtration combined with dialyzer ultrafiltration, and later on due to dialyzer ultrafiltration partly compensated by the capillary absorption of fluid from the tissues). Obviously, beyond this time, the later the RBV monitoring is started, the lower the total RBV reduction will be recorded during dialysis (all curves shown in Figs. 4, 5, 6 continue a linear decrease towards 0 at the end of HD).

As can be seen from Fig. 4, if the HCT measurements are started around 3–5 min after the beginning of the filling procedure, the HCT change observed at the end of HD may be almost 60% higher than the actual HCT change counted from the original patient HCT level (13.5% vs 8.5%). This translates into a circa 50% relative difference in the final RBV decrease calculated from Eq. (1) (− 11.8% vs − 7.8%). For the total blood volume of 5 L this gives an apparent blood volume reduction of circa 600 mL vs 400 mL. If the HCT measurements are started around 3–5 min after the extracorporeal circuit is primed with blood (as recommended in the Crit-Line IV quickstart guide^16^), which in our simulations correspond to 5–7 min after the beginning of the filling procedure, the HCT changes observed during HD will be still overestimated.

Also, depending on the moment of starting the HCT measurements, one may obtain a higher or lower difference between the RBV change calculated from HCT and the actual RBV change counted from the same moment. The maximal difference falls at the end of filling the extracorporeal circuit with the patient’s blood (see Fig. 4), when the total circulating volume stops increasing (due to saline infusion) and starts to decrease (due to increased capillary filtration resulting from the reduced plasma oncotic pressure). The end-dialysis RBV decrease counted from this moment (the highest possible RBV decrease) will not be fully reflected by the HCT-based RBV estimate, because the initial reference HCT will be taken from the not-yet-fully-diluted blood sample appearing in the arterial blood line. This is however less important, given that the whole point of RBV tracking is to capture the RBV changes counted from the state before the dialysis procedure and not from any other moment of time. Therefore, the HCT-based estimate of RBV changes should be only compared to the actual RBV changes counted from the start of the HD procedure (i.e. with respect to the original state of the patient). From this point of view, if the HCT measurements are started within approximately 15 min from the beginning of the whole HD procedure, they will always overestimate the actual RBV changes counted from the very beginning of the procedure with the highest overestimation occurring when the measurements are started within a couple of minutes after the filling procedure is completed.

In Figs. 4, 5, 6 we showed the simulated dialysis-induced relative changes of apparent HCT as a function of the moment of starting HCT measurements during the HD procedure. We did not show the analogous results for HGB or TBP measurements, but as is clear from Fig. 2, the relative changes of HGB and TBP feature exactly the same patterns to those shown for HCT with the same explanation as provided above for HCT. In Fig. 2 one can notice, however, that the RBV changes estimated from HCT variation are always slightly lower than those estimated from HGB or TBP variation. This is due to the fact that during a typical HD the erythrocytes become slightly smaller due to osmotic water shift from erythrocytes to plasma (included in our model), which distorts the RBV estimation and leads to underestimation of the actual RBV changes (the difference between dialysis-induced increase of HCT and that of HGB corresponds approximately to the decrease in the mean volume of erythrocytes). This effect has been confirmed in our previous study in which we analysed clinical data from dialysis patients^22^.

The magnitude of the overestimation of the actual RBV changes by starting HCT measurements at some time after the start of the HD procedure depends on the total volume of the extracorporeal circuit or on the total volume of saline infused to the patient, as shown in Fig. 5. The higher the priming volume, the larger the possible error in estimating RBV changes from a delayed onset of measurements due to the more pronounced diluting effect of the saline. Please note that the same effect would be observed if we would have kept in our simulations a constant priming volume and analysed a smaller patient (i.e. a patient with a lower blood volume). Hence, it is the proportion of the priming volume to the patient size that counts.

Our simulations refer to a patient connected to the dialyzer through an arteriovenous fistula or graft, but the same diluting effect will be observed in a patient with a central venous catheter, except for a marginally longer time it would take the first portion of saline-diluted blood to reach the vena cava compared to reaching the fistula in the arm.

When the priming saline is not infused to the patient but discarded, the possible time-related error in the RBV changes estimated from HCT variation is rather negligible (see Fig. 6). In this case the relatively small differences result from the initial changes in the composition of blood flowing into the dialyzer due to the slightly reduced capillary filtration following the sudden reduction of blood volume circulating within the patient’s body after some of the blood was used to fill the extracorporeal circuit.

Please note that in our simulations we have assumed that at the beginning of the whole HD procedure our virtual patient is in a steady-state condition in terms of fluid and blood distribution. In reality, this may not be the case with the patients presenting internal water and solute imbalances affecting the blood volume and hence affecting RBV changes during dialysis. Note also that the diluting effect of the infused saline seen at the level of arterial blood line could be much higher and faster in the case of access recirculation, which is, however, unlikely with a low dialyzer blood flow rate used for filling the extracorporeal circuit (in this study assumed at 100 mL/min).

Note also that there are various parameters influencing blood volume changes during HD, e.g. ultrafiltration rate or dialyzer blood flow rate (the latter affecting the diffusive solute removal and thus affecting plasma osmolarity and transcapillary fluid exchange). However, our aim was not to study blood volume changes per se but only to investigate the potential error in estimating those changes depending on the moment of starting the measurements of hemoconcentration markers (the aforementioned parameters have no impact on the initial dilution of blood with the priming saline).

Finally, in our simulations we have assumed that when blood fills the extracorporeal circuit, it forces (pushes) all priming saline out of the circuit. In reality, some of the saline may stay in the extracorporeal circuit for a bit longer until it fully mixes with the flowing blood, in particular in the irregularly-shaped blood chamber where the measurements are taken. This is the main reason why the Crit-Line manufacturer recommends to wait 3–5 min before the measurements are started^16^. Additionally, the Crit-Line system features an alert when the HCT changes by more than 5 percentage points in the first 10 min, which indicates a possible transition between saline and blood as seen by the optical sensor in the blood chamber^16^.

In summary, our simulations show that when the priming saline is infused to the dialysis patient, the RBV monitoring through measurements of HCT, HGB or TBP may lead to an overestimation of the actual RBV changes, especially when the priming volume is large with respect to the patient’s size, which is due to the initial dilution of blood by the infused saline and the time it takes for the uniform mixing of blood across the whole circulatory system. Such a possible overestimation is the highest when the measurements are started within a couple of minutes after the filling procedure is completed. Given that the methods of estimating RBV changes during HD are in general subject to several assumptions^22^ and limitations^11–15^, we believe the dialysis staff should be aware of this additional confounding effect as discussed in this paper. This may be also important for research projects studying in detail HD-induced RBV changes and their impact on the cardiovascular system function.

## Data Availability

The detailed description of the mathematical model used in this study as well as the values of all model parameters and initial conditions may be found in our previous work, as mentioned in the manuscript.

## References

[1] Schneditz D (1992). Nature and rate of vascular refilling during hemodialysis and ultrafiltration. Kidney Int..

[2] Pietribiasi M (2015). Kinetics of plasma refilling during hemodialysis sessions with different initial fluid status. ASAIO J..

[3] Pietribiasi M, Wojcik-Zaluska A, Zaluska W, Waniewski J (2018). Does the plasma refilling coefficient change during hemodialysis sessions?. Int. J. Artif. Organs.

[4] Mitsides N, Pietribiasi M, Waniewski J, Brenchley P, Mitra S (2019). Transcapillary refilling rate and its determinants during haemodialysis with standard and high ultrafiltration rates. Am. J. Nephrol..

[5] McIntyre CW, Burton JO (2009). The management of intradialytic hypotension. Brit. J. Ren. Med..

[6] Henrich WL (2008). Intradialytic hypotension: a new insight to an old problem. Am. J. Kidney Dis..

[7] Reeves, P. B., Mc Causland & F, R., Mechanisms, clinical implications, and treatment of intradialytic hypotension. *Clin J Am Soc Nephro* **13**, CJN.12141017 (2018). 10.2215/CJN.12141017 PMC6086712 29483138

[8] Reilly RF (2014). Attending rounds: a patient with intradialytic hypotension. Clin. J. Am. Soc. Nephrol..

[9] Davenport A (2006). Intradialytic complications during hemodialysis. Hemodial Int..

[10] Polaschegg, H. D. & Levin, N. W., in *Replacement of Renal Function by Dialysis*, edited by Hörl, W. H., Koch, K. M., Lindsay, R. M., Ronco, C. & Winchester, J. F. (Springer Netherlands, Dordrecht, 2004).

[11] Dasselaar JJ, Huisman RM, de Jong PE, Franssen CF (2005). Measurement of relative blood volume changes during haemodialysis: merits and limitations. Nephrol. Dial. Transplant.

[12] Dasselaar JJ, van der Sande FM, Franssen CF (2012). Critical evaluation of blood volume measurements during hemodialysis. Blood Purif..

[13] Schneditz D (2016). Concordance of absolute and relative plasma volume changes and stability of Fcells in routine hemodialysis. Hemodial. Int..

[14] Schneditz D, Kron J, Hecking M (2018). Anything goes? High time for smart blood volume monitors. ASAIO J..

[15] Keane DF, Baxter P, Lindley E, Rhodes L, Pavitt S (2018). Time to reconsider the role of relative blood volume monitoring for fluid management in hemodialysis. ASAIO J..

[16] Fresenius Medical Care, Crit-Line IV Monitor. Quickstart guide (2016).

[17] Fresenius Medical Care Renal Therapies Group, LLC, Critl Line IV User's Guide (2017).

[18] Thijssen, S., Raimann, J. G., Usvyat, L. A., Levin, N. W. & Kotanko, P., in *Hemodialysis: New Methods and Future Technology.*, edited by Ronco, C. & Rosner, M. H. (Karger, Basel, 2011), Vol. 171, pp. 84–91.

[19] Pstras L, Waniewski J (2019). Mathematical modelling of haemodialysis: cardiovascular response, body fluid shifts, and solute kinetics.

[20] Pstras L, Thomaseth K, Waniewski J, Balzani I, Bellavere F (2017). Mathematical modelling of cardiovascular response to the Valsalva manoeuvre. Math. Med. Biol..

[21] Pstras L, Thomaseth K, Waniewski J, Balzani I, Bellavere F (2017). Modeling pathological hemodynamic responses to the Valsalva maneuver. J. Biomech. Eng..

[22] Pstras L, Debowska M, Wojcik-Zaluska A, Zaluska W, Waniewski J (2019). Hemodialysis-induced changes in hematocrit, hemoglobin and total protein: Implications for relative blood volume monitoring. PLoS ONE.

